# Association between Exposure to HSV1 and Cognitive Functioning in a General Population of Adolescents. The TRAILS Study

**DOI:** 10.1371/journal.pone.0101549

**Published:** 2014-07-01

**Authors:** Iris Jonker, Hans C. Klein, Hester E. Duivis, Robert H. Yolken, Judith G. M. Rosmalen, Robert A. Schoevers

**Affiliations:** 1 University of Groningen, University Medical Center Groningen, Department of Psychiatry, Interdisciplinary Center for Psychopathology and Emotion regulation (ICPE), Groningen, The Netherlands; 2 Center of Research on Psychology in Somatic Diseases, Department of Medical and Neuropsychology, Tilburg University, Tilburg, The Netherlands; 3 University of Groningen, University Medical Center Groningen, Department of Internal Medicine, Groningen, The Netherlands; 4 Johns Hopkins Medical School, Baltimore, Maryland, United States of America; University of Western Ontario, Canada

## Abstract

**Background:**

Infections with different herpes viruses have been associated with cognitive functioning in psychiatric patients and healthy adults. The aim of this study was to find out whether antibodies to different herpes viruses are prospectively associated with cognitive functioning in a general adolescent population.

**Methods:**

This study was performed in TRAILS, a large prospective general population cohort (N = 1084, 54% female, mean age 16.2 years (SD 0.6)). At age 16, immunoglobulin G antibodies against HSV1, HSV2, CMV and EBV were measured next to high sensitive C-Reactive Protein (hsCRP). Two years later, immediate memory and executive functioning were assessed using the 15 words task and the self ordered pointing task. Multiple linear regression analysis with bootstrapping was performed to study the association between viral infections and cognitive function, adjusting for gender, socioeconomic status, ethnicity, and cannabis use.

**Results:**

Presence of HSV1 antibodies was associated with memory function ((B = −0.272, 95% CI = −0.556 to −0.016, p = 0.047)), while the association with executive functioning did not reach statistical significance (B = 0.560, 95% CI is −0.053 to 1.184, p = 0.075). The level of HSV1 antibodies was associated with both memory function (B = −0.160, 95% CI = −0.280 to −0.039, p = 0.014) and executive functioning (B = 0.296, 95% CI = 0.011 to 0.578, p = 0.046). Other herpes viruses and hsCRP were not associated with cognitive functioning.

**Conclusions:**

Both presence and level of HSV1 antibodies are prospectively associated with reduced cognitive performance in a large cohort of adolescents.

## Introduction

Cognitive impairment is an important clinical problem that accompanies many psychiatric disorders. It has been described in schizophrenia [1] and in bipolar disorder and unipolar depression, both during an episode of depression [2] and in a remitted, euthymic state [3]. In these patients, cognitive impairment is associated with difficulties in social, occupational, and educational functioning and with worse treatment outcomes [1], [4]. Although the factors that cause cognitive impairment are largely unknown, it is suggested that the presence of antibodies to herpes simplex virus 1 (HSV1) may play a role. A recent review summarized several studies that found that HSV1 antibodies were associated with impaired immediate memory and executive functioning in patients with schizophrenia [5]. This association was also found in patients with bipolar disorder [6], [7]. Antibodies to cytomegalovirus (CMV) were found to be associated with impaired visual search and memory function in patients with schizophrenia [8]. Earlier studies did not find an association between cognitive functioning and other herpes viruses such as Epstein Barr virus (EBV) and herpes simplex type 2 (HSV2) [6], [9], [10].

The presence of viral antibodies indicates exposure to these viruses in the past. Theoretically, this association between viral infection and cognitive performance can be explained in two ways. The first explanation is that viral infection leads to persistent infection of the central nervous system (CNS). Human herpes viruses can cause such infections. They have the ability to be latent in the CNS, where they might be re-activated and induce neuroinflammation, which is associated with neurological and psychiatric symptoms [11]. The limbic brain, especially the hippocampus, is found to have specific affinity for HSV1 latency. In post mortem brains of patients with herpes encephalitis, HSV1 was found to be present in the limbic system [12]. The limbic brain, together with the prefrontal cortex, is an important region for cognitive functioning, involved in working memory [13] as well as executive functioning [14]. Imaging studies in patients with schizophrenia showed that cognitive changes as well as psychosis were associated with hippocampal inflammation [15].

A second explanation is that viral infections affect cognitive functioning through systemic inflammation, demonstrated by associations with C-reactive protein (CRP) in elderly [16], in healthy adults [17], in patients with schizophrenia [18] and in patients with major depression [19]. This effect is thought to be caused by vascular damage [20]. The aggregate number of pathogens with which an individual is infected, referred to as pathogen burden, was shown to be more strongly associated with vascular damage than the type of viral agent [21]. A recent study in schizophrenia patients and healthy controls found that the amount of herpes viruses that the participants were exposed to was associated with the reduction of cognitive performance, regardless of diagnosis [22]. These results suggest that systemic inflammation could also be the underlying mechanism causing impaired cognitive functioning.

Thus, psychiatric disorders are often accompanied by cognitive impairment and cognitive impairment is found to be associated with viral antibodies in patients with schizophrenia and in patients with mood disorders. The causal pathway for this association, however, remains unknown. Viral infections might be associated with psychopathology, which in turn causes cognitive impairment. Alternatively, it is possible that viral infections are directly associated with cognitive impairment, which then may or may not be followed by a psychiatric disorder. Studies in healthy individuals are needed to further unravel these pathways. At present, such studies are scarce and findings are inconsistent. One study suggested that there is an association between HSV1 infection and cognitive functioning in healthy adults [10], whereas an earlier study from the same research group in a different sample did not find such an association [23]. Another study investigated the association between cognitive functioning and HSV1 antibodies in both schizophrenia patients and healthy controls. An association was found in the whole group, with no effect of diagnosis on this association [24]. This suggests that this association is also present in healthy participants. Previous studies investigating inflammatory aspects and cognition have mostly been performed in adults. This has the disadvantage that with increasing age, inflammatory and vascular problems increase due to a variety of causes, and it is therefore difficult to study direct effects of viruses on cognition.

The aim of this study is to investigate whether exposure to different herpes viruses, reflected in the presence of viral antibodies, is predictive of cognitive performance, as indicated by short term memory and executive functioning. Our primary hypothesis is that antibodies to latent herpes viruses are associated with cognitive functioning in healthy adolescents. Our secondary hypothesis is that the pathogen burden, the number of different viruses, is more strongly associated with cognitive functioning in healthy adolescents than the exact type of viral agent. We will also test the potential role of systemic inflammation, as reflected in levels of high-sensitive CRP (hsCRP), in the association between viral infection and cognitive function.

We will test our hypotheses in TRAILS, a large prospective general adolescent population cohort with measurements being performed every two years, from age 10 onwards. In this study, we focus on viral antibodies that have been measured at age 16, and cognitive functioning assessed two years later. Because presence of viral antibodies was found to be associated with immediate memory and executive functioning in patients with schizophrenia and bipolar disorder, we focus on these cognitive domains.

## Methods

### Study population

The study is part of the TRacking Adolescents' Individual Lives Survey (TRAILS), a large prospective population study of 2230 Dutch adolescents from the north of the Netherlands examining causes and outcomes of physical and mental health from childhood into adulthood. The survey was approved by the national ethical committee “Centrale Commissie Mensgebonden Onderzoek”. Written Informed Consent was obtained from children and their parents or caretakers. A more elaborate description of the design can be found elsewhere [25]. In short, all 135 primary schools in the five major municipalities in the northern Netherlands were asked to participate. Children with mental retardation, physical incapability or language problems were excluded. The study started in 2001 with the first wave of measurements, with four follow up measurements every two years. During the third assessment wave, a blood sample was obtained for biomarker analyses (mean age  = 16.2, SD = 0.6). At the University Medical Center Groningen, serum was extracted and stored at −80°C until analysis. During the fourth follow up assessment, at the age of approximately 18, participants performed a set of cognitive tasks for neuropsychological evaluation.

### Cognitive symptoms

The cognitive tests we selected for this paper give information about immediate memory and executive functioning, as cognitive performance in these areas was associated with HSV1 infection in earlier studies [8], [9], [26]. The computer version of the Self Ordered Pointing Test was used to measure executive functioning [27]. In this task, the participants have to select a picture from several pictures on the computer screen, which is repeated several times. The participant is not allowed to select the same picture twice or to select the same place on the computer screen twice. The task consists of four parts, with nine or twelve pictures and with abstract or concrete pictures. The most sensitive outcome measure of this task is the error score [28]. We have combined the error scores of the four tasks to get a total error score of this task. To test immediate memory function, we used the Rey's Verbal Learning Test. In this test, participants were asked to memorize 15 words that are read aloud by the task leader, which is repeated five times. We used the total amount of words that the participants could remember at the first three recalls, because previous studies have found ceiling effects when all five trials were used [29]. Verbal task instructions were given before each task. To ensure that the adolescents understood these instructions, practice trials were performed preceding task assessment.

### Measurement of antibodies

Infection of the human body by most infectious agents is associated with the development of persistent immunoglobulin G (IgG) class antibodies that can be measured in the blood years after the infection. IgG serum antibodies to different agents of the Herpes virus family (HSV1, HSV2, CMV and EBV) were measured using solid-enzyme immunoassay methods [30]. For each assay, a result was defined as positive or negative based on comparison with the reactivity of specific antibody standards saved along with the blood samples. These standards consisted of samples with predefined levels of reactivity of specific herpes antigens [31].These analyses provided information on the presence of the different antibodies as well as on the level of infectious activity reflected in the levels of the different antibodies. Presence of antibodies reflects whether the adolescent was ever exposed to the virus, whereas levels of antibodies reflect the activity of the virus at age 16.The aggregate number of different pathogens that the adolescents had antibodies to, was referred to as pathogen burden [21]. All serological tests were carried out by standard procedure at the Stanley Laboratory of Developmental Neurovirology, Baltimore, Maryland.

### Measurement of hsCRP

C-reactive protein (CRP) is produced by the liver in reaction to pro-inflammatory cytokines and is a measurement for systemic inflammation. High sensitive-CRP (hsCRP) is more sensitive to small changes in systemic inflammation, since it is detected at lower values [32]. hsCRP was determined using a immunonephelometric method with a lower detection limit of 0.175 mg/L. Intra-assay coefficients of variance ranged from 2.1 to 4.4, and inter-assay coefficients of variation coefficients of variance ranged from 1.1 to 4.0.

### Confounders

Socioeconomic status (SES) was defined by family income, educational level of the father and the mother, and occupational level of both parents using the International Standard Classification of Occupations [33]. A SES variable was created by averaging the indicators after standardization. Factor analysis in this population showed a Crohnbach's alpha of 0.84 [34]. Affective symptoms were assessed using the Affective Problems scale of the Youth Self-Report (13 items, Cronbach's alpha  = 0.75) [35]. The frequency of cannabis use in the four weeks prior to the cognitive test was assessed in a self-report questionnaire; answers were categorized into use of cannabis or no use of cannabis. Adolescents who had at least one parent born in a non-western country were identified to estimate ethnicity; this included Turkey, Morocco, Surinam, Dutch Antilles, Indonesia, Iraq, Iran, Somalia, etc.

### Statistical analysis

Because of non-normal distribution of hsCRP, Mann Whitney U-tests were used to examine the association between hsCRP values and the presence of viral antibodies. To determine the association between hsCRP and pathogen burden, Spearman's rho was used, because both hsCRP and pathogen burden had a non-normal distribution. Multiple linear regression analysis was conducted to evaluate whether hsCRP, the presence and the levels of viral antibodies, and the pathogen burden were associated with cognitive functioning two years later. Because of non-normal distribution of data, we performed bootstrapping analysis in our regression model. Bootstrapping gets around the problem of non-normal distribution by estimating the properties of the sampling distribution from sample data (called bootstrapping samples). The values of the samples are used to estimate the limits of the 95% confidence interval of the parameter. An effect is considered significant when the bootstrap 95%-CI does not include zero [36]. In the multiple regression models, we adjusted for the following confounders, known to be associated with our predictor (pathogen burden, viral infections or CRP) and our outcome measure (cognitive functioning): gender [37]–[39], socioeconomic status (SES) [39]–[41], ethnicity [42], [43] and cannabis use [44], [45]. Affective symptoms are also associated with cognitive functioning and inflammatory measurements [46], [47]. It is, however, unknown whether they would be influencing outcomes as a confounder or as a mediator. Therefore, we repeated analysis with affective symptoms as a covariate, to perform a sensitivity analysis.

## Results

### Population Characteristics

**Figure 1:**
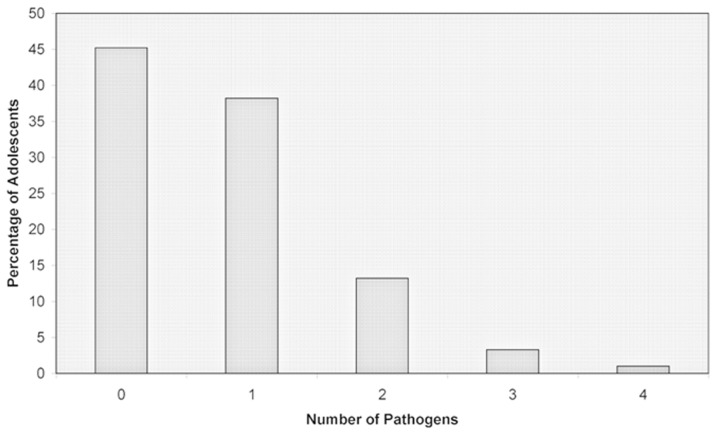
Pathogen Burden. Distribution of number of pathogens in percentages, present in the adolescent population.

The population characteristics are summarized in table 1. Pathogen Burden is shown in figure 1.

**Table 1:** Population Characteristics.

General Characteristics	% (n)
Age (mean, SD)	16.2 (0.6)
Female	54.0 (585)
SES (Median, IQR)	0.15 (−0.4, 0.8)
Dutch Ethnicity	90.4 (980)
Cannabis use	17.7 (192)
hsCRP (ug/l) (Median, IQR)	0.4 (0.2, 1.0)
**Prevalence of antibodies**	
HSV1	24.4 (264)
HSV2	1.2 (13)
EBV	24.6 (267)
CMV	24.7 (268)
**Cognitive Performance**	
SOPT error scores (Median, IQR)	8.0 (6.0, 10.0)
15 words immediate memory (Median, IQR)	9.0 (7.7,10.3)

Numbers are presented as %(N) unless otherwise specified.

IQR  =  Interquartile range, SES  =  socio-economic status, hsCRP  =  high sensitive C-Reactive Protein, HSV1  =  Herpes Simplex Virus 1, HSV2  =  Herpes Simplex Virus 2, EBV  =  Epstein Barr Virus, CMV  =  Cytomegalovirus, SOPT  =  self ordered pointing task.

### Association between viral antibodies and cognitive functioning

Table 2 summarizes the association between the presence and the levels of the different viral antibodies and total error scores on SOPT. Table 3 summarizes the association between the presence and the levels of the viral antibodies and immediate memory scores. The presence of HSV1 antibodies was associated with the immediate memory score of the 15 words task, but not with the total error score of SOPT. The level of HSV1 antibodies was associated with both the 15 words and with the error score of the SOPT. Both presence and levels of antibodies to the other viruses were not significantly associated with any of the measures of cognitive performance. Analyses were repeated with affective symptoms as covariate, yielding essentially the same results.

**Table 2:** Association between the presence and levels of viral antibodies and executive functioning two years later, as measured by Self Ordered Pointing Task.

	Presence of antibodies	Levels of antibodies
HSV1	B = 0.554, 95% CI: −0.074 to 1.178, p = 0.078	B = 0.295, **95% CI: 0.008 to 0.576, p = 0.045**
HSV2	B = 0.158, 95%CI: −1.000 to 1.221, p = 0.785	B = 0.327, 95%CI: −0.317 to 1.098, p = 0.332
EBV	B = −0.209, 95% CI: −0.746 to 0.386, p = 0.460	B = −0.026, 95% CI: −0.467 to 0.410, p = 0.908
CMV	B = 0.471, 95% CI: −0.120 to 1.161, p = 0.158	B = 0.155, 95% CI: −0.123 to −0.486, p = 0.324

HSV1  =  Herpes Simplex Virus 1, HSV2  =  Herpes Simplex Virus 2, EBV  =  Epstein Barr Virus, CMV  =  Cytomegalovirus.

Outcomes of multiple regression analyses with bootstrapping, adjusted for gender, SES, ethnicity, and cannabis use.

**Table 3:** Association between the presence and levels of viral antibodies and immediate memory two years later, as measured by the immediate recall part of the 15 words task.

	Presence of antibodies	Levels of antibodies
HSV1	B = −0.273, **95%CI:−0.522 to −0.015, p = 0.046**	B = −0.160, **95%CI:−0.728 to −0.041, p = 0.015**
HSV2	B = −0.541, 95%CI: −1.137 to 0.281, p = 0.208	B = −0.224, 95%CI: −0.728 to 0.193, p = 0.330
EBV	B = −0.052, 95%CI: −0.302 to 0.214, p = 0.701	B = −0.098, 95%CI: −0.296 to 0.110, p = 0.356
CMV	B = 0.149, 95%CI: −0.072 to 0.400, p = 0.234	B = 0.083, 95%CI: −0.026 to 0.198, p = 0.168

HSV1  =  Herpes Simplex Virus 1, HSV2  =  Herpes Simplex Virus 2, EBV  =  Epstein Barr Virus, CMV  =  Cytomegalovirus.

Outcomes of multiple regression analyses with bootstrapping, adjusted for gender, SES, ethnicity, and cannabis use.

### Association between Pathogen Burden and cognitive functioning

The immediate memory score of the 15 words task was not associated with Pathogen Burden (B = −0.058, 95% CI is −0.186 to 0.086, p = 0.425). Likewise, the total error score of the SOPT was not associated with Pathogen Burden (B = −0.468, 95% CI is −0.084 to 0.548, p = 0.145). We repeated the analyses with affective symptoms as covariate, which gave essentially the same results.

### Inflammation and viral infection

We performed Mann Whitney U tests to assess whether the presence of viral antibodies was associated with systemic inflammation, as reflected in levels of hsCRP. The presence of HSV1 antibodies was significantly associated with hsCRP: the HSV1 positive group (mean rank 592.79) had significantly higher levels of hsCRP than the HSV1 negative group (mean rank 523.67) (p = 0.001). Presence of antibodies to other viruses was not associated with hsCRP levels. Pathogen burden was also associated with hsCRP (Spearman's rho = 0.090; p = 0.003).

### Association between systemic inflammation and cognitive functioning

Multiple regression analysis, adjusting for gender, SES, ethnicity and cannabis use, was performed to test the association between the scores on the two different cognitive tasks and the levels of hsCRP. The immediate memory score of the 15 words task was not associated with hsCRP levels (B = −0.002, 95% CI is −0.024 to 0.015, p = 0.817). Likewise, the total error score of the SOPT was not associated with the hsCRP levels (B = −0.013, 95% CI is −0.052 to 0.018, p = 0.331). We repeated the analyses with affective symptoms as covariate, which gave essentially the same results. Thus, hsCRP does not suffice with the conditions to be a mediator in the relationship between HSV1 antibodies and cognitive functioning.

## Discussion

This is, to our knowledge, the first prospective study examining the association between viral antibody status and subsequent cognitive function in a large cohort of healthy adolescents. Our findings showed an association between the presence and levels of HSV1 antibodies at age 16 and cognitive function at age 18, indicated by performance at a short term memory task and an executive functioning task. There was no association between cognitive function and CRP, other viruses, or total pathogen burden.

A major strength of this study is the size of our study population, enabling an accurate estimation of the temporal association between viral antibodies and cognitive performance two year afterwards. A second strength is the fact that we studied cognitive performance in healthy adolescents; at this young age cognitive performance is not yet influenced by other factors such as vascular damage and normal aging. Some limitations of our study need to be addressed as well. One limitation is the fact that antibody status does not inform us on the moment in their life at which the adolescents were exposed to the herpes viruses. Also, viral antibodies were assessed at age 16, while cognitive function was measured at age 18. It is likely that virus burden was increased between age 16 and 18, especially since kissing behavior is an imported way to get infected with herpes viruses. The prevalence of the herpes viruses at age 16 in our population was low compared to findings in adolescents in age group 15–19 from other countries, with 70% seroprevalence for EBV in the USA [48], 1.6% for HSV2, 39% for HSV1 in the USA [49], and 35% for CMV in Germany [50]. Factors associated with EBV seropositivity include ethnicity, crowdedness, household income, household education level and health insurance status [48], and international variability in these factors might contribute to our low EBV seroprevalence. Nevertheless, this low prevalence facilitates the testing of differences in cognitive performance in adolescents that are exposed to the viruses and adolescents that are not. Another limitation is that cognitive functioning was not assessed at age 16, making it impossible to calculate an estimate of cognitive change. Furthermore, a limitation is that most adolescents had no antibodies to pathogens, or only antibodies to one pathogen. This probably makes pathogen burden per se a less informative measurement in this age group.

We were the first to find a significant association between the presence and levels of viral antibodies at age 16 and cognitive performance two years later. The association between HSV1 and cognitive performance is in line with findings reported by previous studies using a cross-sectional design [5], [10]. Our findings could mean that the association between antibodies and cognitive functioning is not a state effect, but that the presence of antibodies has longer-lasting effects on cognitive functioning. We used different tasks to test the cognitive domains that were found to be associated with HSV1 in previous studies [5], [7]. The fact that we found an association in the same cognitive domain as in previous research with a different task makes the finding more robust. HSV2, EBV and CMV were not associated with cognitive functioning in our study, which is in agreement with previous findings [6], [9], [10]. In contrast to our findings, one previous study found CMV antibodies to be associated with cognitive performance in patients with schizophrenia [8]. In this study, cognitive performance was assessed using the Trail Making Task, which measures attention and task switching. This could be an explanation for the different findings. In a recent case study, two adolescent homozygote twin pairs were followed, in which one of the pair was seropositive for CMV and the other seronegative. In both pairs, there was no difference in neurocognitive development between the seropositive and the seronegative twin [51]. Together with our findings, this suggests that CMV is not predictive for cognitive performance in adolescents.

We found an association between HSV1 antibodies and cognitive function in a healthy population, suggesting that this association is independent of psychopathology. The association between HSV1 antibodies and cognitive functioning was initially studied in patients with mental disorders, because schizophrenia has been shown to be associated with viral infections [52]. In a systematic review on cognitive functioning in schizophrenia, it was hypothesized that cognitive impairment could be considered a risk factor for developing psychopathology [1]. This could mean that HSV1 infection may lead to the development of psychopathology via cognitive impairment.

As discussed in the introduction, the association between viral antibodies and cognitive functioning can be explained by either local infection of the CNS or systemic inflammation leading to toxic effects and vascular damage. Our findings do not differentiate between these explanations. It is, however, remarkable that only presence of HSV1 antibodies was significantly associated with hsCRP and that the presence and level of only the HSV1 antibodies were predictive for cognitive performance. This is in line with a recent study in patients with schizophrenia, in which both high CRP values and HSV1 antibodies predicted cognitive functioning. This association was most pronounced when both HSV1 and high CRP levels were present, but an interaction effect between CRP and HSV1 was not found [53]. The fact that the level of antibodies to HSV1 was more strongly associated with cognitive performance than the presence of antibodies to HSV1 fits both theories. The level of HSV1 antibodies can reflect the activity of the HSV1 virus or the activity of the immune system in general.

In conclusion, our results suggest that previous HSV1 infection, as reflected in the presence of HSV1 antibodies, is predictive for cognitive performance two years later in healthy adolescents. It would be of interest to replicate this study with more frequent measurements regarding cognitive functioning and viral status. Furthermore, herpes related cognitive impairment may also be predictive of the development of mental disorders, the investigation of which would need prospective studies with longer follow-up times. The pathophysiological explanation for the association between HSV1 infection and activity and cognitive functioning currently remains unknown. As temporal lobe structures such as hippocampus are primary hosts for HSV1 in the brain, future studies could be directed towards assessment of viruses or inflammation in this region, for example by using molecular neuro-imaging techniques of the brain.
